# Degenerin channel activation causes caspase‐mediated protein degradation and mitochondrial dysfunction in adult *C. elegans* muscle

**DOI:** 10.1002/jcsm.12040

**Published:** 2015-06-04

**Authors:** Christopher J. Gaffney, Freya Shephard, Jeff Chu, David L. Baillie, Ann Rose, Dumitru Constantin‐Teodosiu, Paul L. Greenhaff, Nathaniel J. Szewczyk

**Affiliations:** ^1^ MRC/ARUK Centre for Musculoskeletal Ageing Research, Faculty of Medicine and Health SciencesUniversity of NottinghamNottinghamNG7 2UHUK; ^2^ Department of Molecular Biology and BiochemistrySimon Fraser UniversityBurnabyBCV5A 1S6Canada; ^3^ Department of Medical GeneticsUniversity of British ColumbiaVancouverBCV6T 1Z4Canada

**Keywords:** Caspase, *C. elegans*, Degenerin, Mitochondria, Muscle

## Abstract

**Background:**

Declines in skeletal muscle structure and function are found in various clinical populations, but the intramuscular proteolytic pathways that govern declines in these individuals remain relatively poorly understood. The nematode *Caenorhabditis elegans* has been developed into a model for identifying and understanding these pathways. Recently, it was reported that UNC‐105/degenerin channel activation produced muscle protein degradation via an unknown mechanism.

**Methods:**

Generation of transgenic and double mutant *C*. *elegans*, RNAi, and drug treatments were utilized to assess molecular events governing protein degradation. Western blots were used to measure protein content. Cationic dyes and adenosine triphosphate (ATP) production assays were utilized to measure mitochondrial function.

**Results:**

*unc‐105* gain‐of‐function mutants display aberrant muscle protein degradation and a movement defect; both are reduced in intragenic revertants and in *let‐2* mutants that gate the hyperactive UNC‐105 channel. Degradation is not suppressed by interventions suppressing proteasome‐mediated, autophagy‐mediated, or calpain‐mediated degradation nor by suppressors of degenerin‐induced neurodegeneration. Protein degradation, but not the movement defect, is decreased by treatment with caspase inhibitors or RNAi against *ced‐3* or *ced‐4*. Adult *unc‐105* muscles display a time‐dependent fragmentation of the mitochondrial reticulum that is associated with impaired mitochondrial membrane potential and that correlates with decreased rates of maximal ATP production. Reduced levels of CED‐4, which is sufficient to activate CED‐3 *in vitro*, are observed in *unc‐105* mitochondrial isolations.

**Conclusions:**

Constitutive cationic influx into muscle appears to cause caspase degradation of cytosolic proteins as the result of mitochondrial dysfunction, which may be relevant to ageing and sarcopenia.

## Background

Maintained muscle protein homeostasis (proteostasis) occurs through intricate regulation of balanced rates of protein synthesis and degradation. Proteostasis is required to maintain contractile ability and muscle as a metabolic reservoir.^1^ Declines in skeletal muscle mass, structure and function are associated with ageing (sarcopenia), cancer (cachexia), COPD, heart failure, and diabetes,^2^ but the proteolytic pathways that govern declines in each of these conditions remain relatively poorly understood.

*Caenorhabditis elegans* is an established laboratory animal in which genetics and genomics can be used to understand physiology and is the animal for which we know the most about genes controlling muscle protein degradation.^3^ In *C*. *elegans* muscle, increased traffic to the proteasomes is normally prevented by neuronal release of acetylcholine^4^ to presumably affect intramuscular calcium signalling^5^ and appears to occur in response to starvation,^6^ denervation,^4^ and neurodegeneration.^7^ Increased traffic to lysosomes via autophagy is controlled by a balance of constitutive fibroblast growth factor and insulin/insulin like growth factor^8^; roughly two dozen other protein kinases appear to oppose autophagic degradation.^9^ These signals converge, modulating activation of autophagy via mitogen‐activated protein kinase (MAPK),^10^ and appear relevant to a model of neurodegeneration^11^ and to ageing.^12^ Lastly, calpains are activated in response to muscle attachment complex disruption, which appears required to maintain muscle in response to use.^13^, ^14^

Recently, *unc‐105* gain‐of‐function mutants were reported to display increased muscle protein degradation via an unknown mechanism.^5^ *unc‐105* encodes a putative mechano‐sensitive ion channel of the ENaC/degenerin family.^15^ ENaC/Degenerin channels are implicated in the pathophysiology of some human neurodegenerative diseases,^16^ and hyperactivity of other ENaC/Degenerin channels causes necrotic‐like neuronal cell death in *C*. *elegans.* 17, 18, 19, 20 Dominant gain‐of‐function mutations in *unc‐105* have previously been shown to cause worms to be small, hypercontracted, and paralyzed.^21^ These phenotypes are suppressed in *unc‐105*; *let‐2* double mutants.^15^ LET‐2 is an alpha‐2 type IV basement membrane collagen^22^ that has been proposed to physically gate the mutationally activated UNC‐105 channel in the muscle plasma membrane and thereby relieve the anomalous ion influx and rescue the aforementioned phenotypes.^15^, ^23^

Here, we report that *unc‐105* mutants but not *unc‐105*; *let‐2* double mutants display caspase‐dependent muscle protein degradation and loss of normal muscle mitochondrial architecture, mitochondrial membrane potential, and maximal mitochondrial ATP production capacity. These changes are associated with decreased mitochondrial‐associated CED‐4. CED‐4, or cell death abnormal protein 4, is the *C*. *elegans* orthologue of mammalian apoptotic protease activating factor 1 (APAF1) and *in vitro* CED‐4 is sufficient to activate the caspase CED‐3. The discovery of CED‐3‐mediated and CED‐4‐mediated programmed cell death in *C*. *elegans* was the subject of the 2002 Nobel Prize in Physiology or Medicine as programmed cell death is now known to be crucial for regulating cell number. Indeed, lack of proper activation of cell death is associated with cancer, autoimmune disease, and neurodegenerative disease. Collectively, our data suggest that excessive cationic influx into muscle leads to pathological changes in mitochondrial architecture and function, and subsequent caspase activation. These data also complete our preliminary understanding of the regulation of activation and increased trafficking to the four major proteolytic systems in *C*. *elegans* muscle, and enable direct testing of the relevance of all four systems to various (patho)physiologic conditions.

## Materials and methods

### Strains and culture

Strains of *C*. *elegans* were handled, maintained, and age‐synchronized as described.^6^ RNAi was as described^13^ using sequence‐verified RNAi (Source BioScience LifeSciences Ltd.). Strains for protein degradation, maximal rates of ATP production, and mitochondrial membrane potential assays are as follows: PD55: *ccls55* V, CC10: *unc‐105*(*n490*) II; *ccls55* V, and CC7: *unc‐105*(*n490*) II; *ccls55* V, *lon‐2*(*e678*) *let‐2*(*n821*) X. Strains for Mitotracker® and Caspase 3/7 substrate assays are as follows: CB5600: *ccls4251* (*myo‐3::Ngfp‐lacZ, myo‐3::Mtgfp*) I; *him‐8*(*e1489*) IV, CC62: *ccls4251* (*myo‐3::Ngfp‐lacZ, myo‐3::Mtgfp*) I; *unc‐105*(*n490*) II; *him‐8*(*e1489*) IV and CC63: *ccls4251* (*myo‐3::Ngfp‐lacZ, myo‐3::Mtgfp*) I; *unc‐105*(*n490*) II; *him‐8*(*e1489*) IV; *lon‐2*(*e678*) *let‐2*(*n821*) X.

### Mitochondrial function

MitoTracker® chloromethyl‐X‐rosamine (CMXRos) *in vivo* accumulation assay. Synchronized worms were grown to early adulthood. Twenty animals per trial were processed as described in 4.7 μM CMXRos.^24^

JC‐10 *in vivo* accumulation assay. Synchronized worms were grown from the L1 stage in the presence of 83 μM JC‐10 (Enzo Life Sciences, NY, USA).

JC‐1 Fluorescence‐activated cell sorting (FACS). Animals were cultured and mitochondria isolated as described.^14^ Mitochondria were incubated in the dark for 15 min at 37°C in 20 μM glutamate, 2 mM malate, 6 μM JC‐1 (Invitrogen, UK). Mitochondria were centrifuged (10 000 g, 5 min), resuspended, and sorted in a Beckman Coulter FC500 flow cytometer (Ex 490 nm and Em 605 nm). Loss of JC‐1 in response to loss of membrane potential was confirmed by addition of 1 μM CCCP.

Maximal rates of ATP production (MRAP). Maximal rates of ATP production (MRAP) were assessed as described.^14^ Mixed‐stage populations were used to obtain sufficient quantity of mitochondria as *unc‐105* mutants are very sick, taking at least 2–3 weeks to produce a population capable of exhausting food supplies on a plate.

### Western blotting

β‐Galactosidase westerns were performed as described^13^ with quantification in Image J. CED‐4,^25^ cytochrome C,^26^ and ATP‐synthase^27^ antibodies were previously validated. Anti‐CED‐4 (Santa Cruz Biotechnology, USA), was used at a 1:100 dilution in 5% milk TBS‐T with secondary (Santa Cruz Biotechnology, USA) at a 1:10 000 dilution. Anti‐cytochrome C and anti‐ATP‐synthase (anti‐ATP5A) (Abcam®, UK) were used at a 1:1000 and 1:2500 dilution in 3% milk TBS, respectively, with secondary (Sigma‐Aldrich®, USA) at a 1:10 000 dilution.

### Measurements of caspase activation

Synchronized adult worms were grown in the presence of 200 μM Z‐DEVD‐ProRed™ (AAT Bioquest, UK) at 20°C and analysed at *t* = 0, 12, 24, and 48 h with red fluorescence noted at all three later time points. For quantification of caspase activity, untreated animals were washed from single plates, homogenized in buffer containing 1% Triton, incubated with the caspase reagent buffer (Caspase‐Glo® 3/7 Assay Kit, Promega, UK) for 45 min at 37°C, and luminescence measured. Activity is expressed versus a standard curve of titrated recombinant human caspase 3 (Promokine, Germany).

### Assessment of β‐galactosidase activity

Animals were stained for β‐galactosidase activity as described.^6^ Control animals (PD55) or *unc‐105* (CC10 on standard *Escherichia coli* strain OP50 in the RNAi conditions) were utilized.

### Whole genome sequencing

Genomic DNA was prepared as described^28^ and sequenced using Illumina Solexa GAII (BC Cancer Agency Genomic Sciences Centre). Sequences were aligned to *C*. *elegans* genome version WS200 using Burrows‐Wheeler Aligner (BWA)^29^ under default settings. Resulting BAM files were processed to identify single nucleotide variants (SNVs) and small insertions or deletions (indels) using VarScan.^30^ Variants were matched to WS200 annotation using CooVar^31^ and categorized as ‘nonsense’, ‘missense’, ‘synonymous’, ‘non‐coding’, ‘frame preserving indel’, or ‘frame shifting indel’. Non‐coding mutations were defined based upon intron and intergenic regions. Only homozygous mutations (a mutation with more than 90% read support for the change) were considered.

### Movement assays

Movement was measured as described.^10^

### Microscopy and statistics

Microscopy. All images were captured as described.^13^ Image analysis and figure preparation was conducted in GIMP and Photoshop.

Statistical analysis. All data are presented as means ± SEM from at least three replicates unless otherwise stated. Statistical differences were assessed using either one‐way ANOVA with Newman–Keuls corrections or two‐way ANOVA with Bonferroni corrections. Significance was determined as *P* < 0.05, and all statistics were completed using GraphPad Prism (USA).

## Results

### Activation of UNC‐105 causes muscle protein degradation

**Figure 1:**
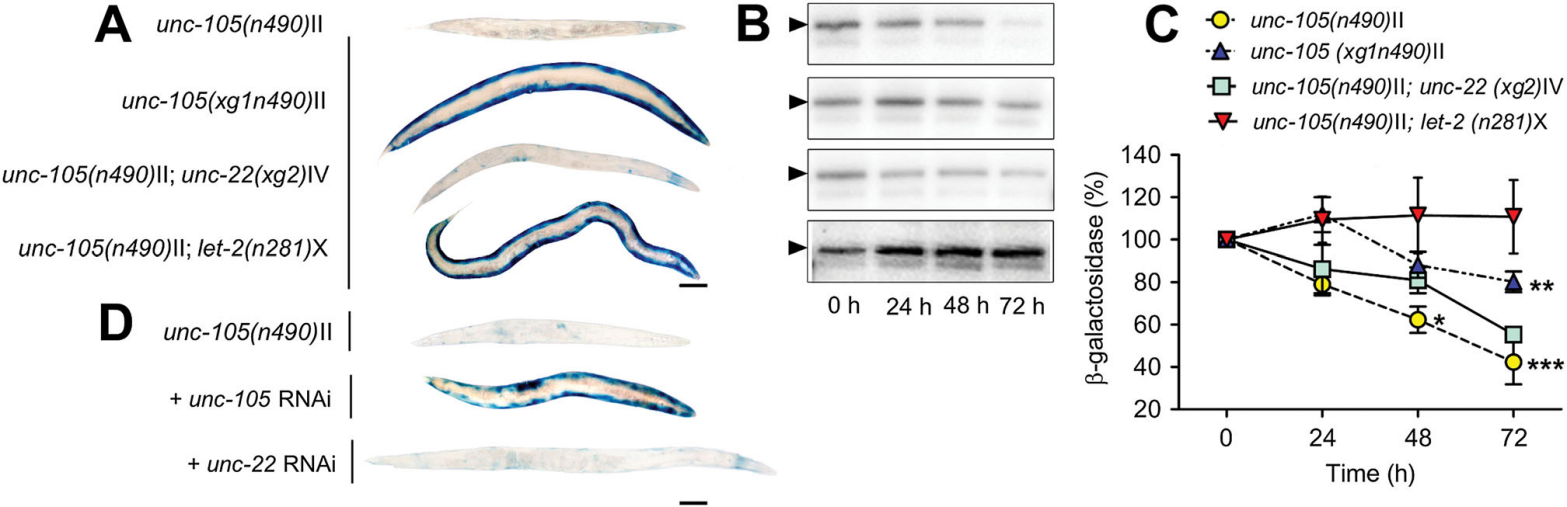
Activation of UNC‐105 causes protein degradation in muscle. Synchronized young adults were used (*t* = 0 h), and all experiments repeated at least three times. (A) Representative stains for β‐galactosidase at *t* = 72 h post‐adulthood; scale bar represents 100 µm. (B) Representative western blots of β‐galactosidase in 30‐worm lysates. Arrows show β‐galactosidase fusion protein at 146 kD. Wild‐type controls are not included as *unc‐105*; *let‐2* results are not significantly different from published wild‐type values.^4^, ^8^, ^10^, ^13^ (C) Kinetics of β‐galactosidase degradation, quantified from three independent western blots. * denotes *P* < 0.05; ** denotes *P* < 0.01; *** denotes *P* < 0.001 between *unc‐105* and *unc‐105*; *let‐2*, two‐way repeated measures ANOVA with Bonferroni correction. (D) Representative stains for β‐galactosidase at *t* = 72 h post‐adulthood; scale bar represents 100 µm.

Recently, it was reported that UNC‐105 activation appears to cause muscle protein degradation that is not suppressed by treatment with proteasome inhibitors.^5^ The time dependent loss of a transgenic β‐galactosidase reporter protein's activity suggests that muscle protein degradation is occurring (*Figure* 1A). This transgenic reporter protein has been shown to be fully stable in muscle of well fed adult worms for at least 72–96 h post‐adulthood but is degraded upon activation of various proteolytic systems.^6^, ^8^, ^13^ To confirm that loss of enzymatic activity was due to degradation, we performed western blots (*Figure* 1B and C). RNAi against *unc‐105* in wild‐type animals had no effect on proteostasis,^5^ while the dominant gain‐of‐function allele *n490* caused protein degradation (*Figure* 1); these observations suggest that it is the activation of the UNC‐105 ion channel that induces muscle protein degradation. In support of this suggestion, we found that RNAi against *unc‐105* attenuates the loss of β‐galactosidase activity in *unc‐105(n490)* mutants (*Figure* 1D).

Previous work has shown that excessive Na^+^ influx in mutants containing another dominant gain‐of‐function mutation in *unc‐105*, *n506*, causes sustained muscle depolarisation that is attenuated in *unc‐105*(*n506*) mutants containing a second mutation in *let‐2.* 23 LET‐2 is an alpha‐2 type IV basement membrane collagen^22^ and treatment of *unc‐105*; *let‐2* double mutants with collagenase restores high levels of Na^+^ influx. These past results suggest that LET‐2 has a physical interaction with UNC‐105, which gates the channel.^23^ Therefore, to further test if UNC‐105 activation leads to increased muscle protein degradation, we tested if protein degradation was suppressed in *unc‐105(n490)*; *let‐2(n821)* double mutants. As shown in *Figure* 1, protein degradation was suppressed in *unc‐105*; *let‐2* double mutants, further suggesting that hyperactivity of the UNC‐105 channel causes muscle protein degradation.

While conducting this work, two spontaneous mutants that resulted in increased population growth rates in the *unc‐105*(*n490*) strain were isolated; designated as *xg1* and *xg2* (*Figure* S1). *xg1* mutants display reduced muscle protein degradation, while *xg2* mutants do not (*Figure* 1). As *unc‐105* mutants pick up suppressing mutations at a high rate,^32^ we presumed that *xg1* and *xg2* might be mutations in known suppressing genes. Previously identified suppressors of *unc‐105* include mutations in *unc‐15*, *unc‐22*, *unc‐54*, *unc‐96*, *crt‐1*, *let‐2,* and *unc‐105* itself^15^, ^19^, ^32^, ^33^, ^34^; these genes encode paramyosin, twitchin, myosin heavy chain A, a Lin11, Isl‐1 & Mec‐3 (LIM) domain containing protein that localizes to M‐lines, calreticulin, a collagen, and a degenerin channel, respectively. Because *xg1* looked wild‐type and the most frequently isolated mutation that suppresses *unc‐105* with a wild‐type appearance was *unc‐105*,^32^, ^33^ we presumed that *xg1* might be an intragenic revertant. Similarly, as *xg2* visibly twitched and *unc‐22* is the only isolated suppressor that twitches,^32^ we presumed that *xg2* might be an allele of *unc‐22*. Full genome sequencing (*Figure* S2, *Table* 1, Dataset S1) confirmed a 4bp insertion *unc‐105* at II: 8118943 in the strain containing *xg1* and a nonsense mutation disrupted *unc‐22* at IV: 11984751 in the strain containing *xg2*. RNAi against *unc‐105* or *unc‐22* produces the same effect on β‐galactosidase activity in *unc‐105(n490)* mutants (*Figure* 1D) as does allele *xg1* or *xg2* (*Figure* 1A). In the intragenic revertant, *xg1*, a premature stop codon is predicted, like RNAi against *unc‐105*, to reduce the abundance of UNC‐105. The decreased abundance of activated UNC‐105 presumably results in less Na^+^ influx, which suggests that hyperactivation of the channel causes muscle protein degradation.

**Table 1:** Summary of single nucleotide variations and indels in strains CC24 (containing *xg1*) and CC50 (containing *xg2*)

Strain	Synonymous	Silent	Missense	Nonsense	Frame‐preserving indel	Frame‐shifting indel
CC24	4	47	4	1	0	2
CC50	1	20	2	1	0	0

### Muscle protein degradation in response to UNC‐105 activation is distinct from previously described mechanisms

**Figure 2:**
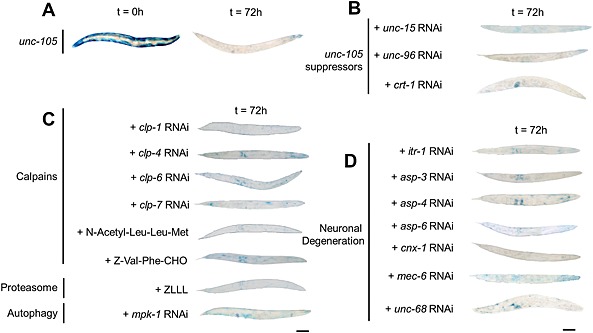
UNC‐105‐induced protein degradation is not suppressed by known suppressors of protein degradation or neurodegeneration in *Caenorhabditis elegans*. Synchronized young adults were used (*t* = 0 h), and all experiments repeated at least three times. Representative stains for β‐galactosidase at *t* = 72 h post‐adulthood; scale bar represents 100 µm. (A) Untreated *unc‐105* mutants. (B) RNAi against known suppressors of *unc‐105* growth or movement defect. (C) RNAi or drugs targeting known components of calpain,^13^ proteasome,^4^, ^6^ or autophagic^8^, ^10^ muscle protein degradation in *C*. *elegans*. (D) RNAi against known suppressors of MEC‐4 degenerin‐induced neuronal degeneration.

**Figure 3:**
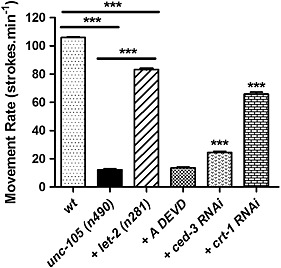
Caspase inhibition does not markedly restore the movement defect in *unc‐105* mutants. Synchronized young adults were used (*t* = 0 h), and all experiments repeated at least three times. Movement of the head back and forth in liquid was determined for 10 adults each assessed 10 times. *** denotes *P* < 0.001, two‐way ANOVA with Bonferroni correction.

As *unc‐105*, other degenerin channels, and muscle protein degradation have all previously been studied in *C*. *elegans*, we were curious if other known suppressors of *unc‐105* gain‐of‐function, mutations that reduce neuronal cell death in response to hyperactivity of other ENaC/Degenerin channels,^17^, ^18^, ^19^, ^35^ and/or suppression of previously identified proteolytic pathways could attenuate the protein degradation induced by UNC‐105 activation (*Figure* 2A). RNAi against *unc‐15*, *unc‐96*, and *crt‐1* each also failed to suppress the protein degradation observed in *unc‐105* gain‐of‐function mutants (*Figure* 2B). The failure to suppress degradation was not simply as the result of ineffective RNAi as RNAi against *crt‐1* improved the movement defect in *unc‐105* gain‐of‐function mutants (*Figure* 3), as expected.^19^ RNAi against *itr‐1*, *asp‐3*, *asp‐4*, *asp‐6*, *cnx‐1*, *mec‐6*, or *unc‐68*, also each failed to block protein degradation (*Figure* 2D); these genes encode the following: inositol trisphosphate receptor, three aspartyl proteases, calnexin, a degenerin channel subunit, and ryanodine receptor. Lastly, as shown in *Figure* 2C, protein degradation in response to UNC‐105 activation is not suppressed by treatment with proteasome inhibitor, calpain inhibitor or RNAi against calpains, or by RNAi against *mpk‐1* that acts to control autophagic degradation.

### Caspases are activated in response to UNC‐105 activation

**Figure 4:**
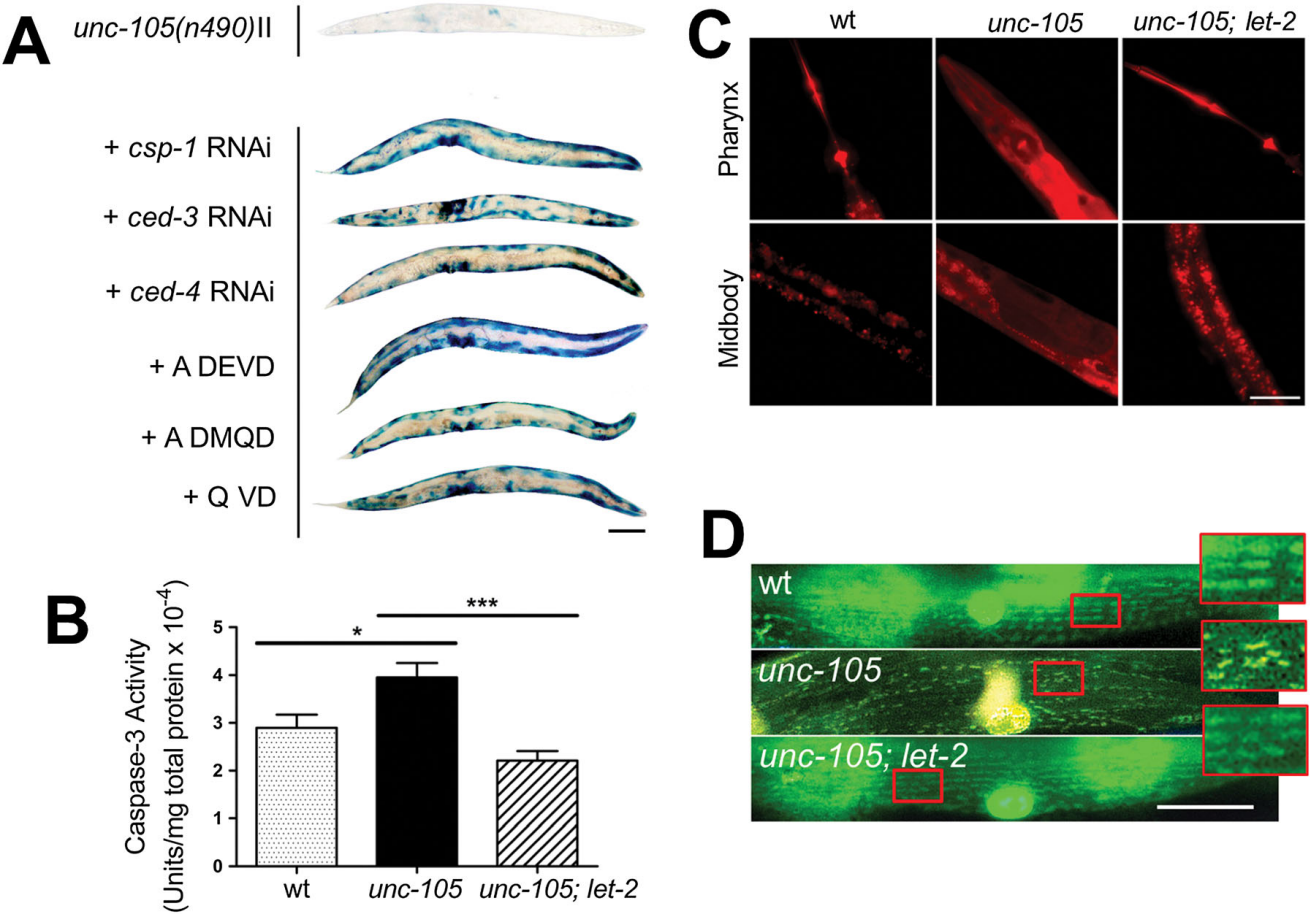
Caspases are activated in muscles of *unc‐105* mutants. Synchronized young adults were used (*t* = 0 h), and all experiments repeated at least three times, for (B) seven independent experiments. (A) Representative stains for β‐galactosidase at *t* = 72 h post‐adulthood in *unc‐105* mutants either untreated or treated with RNAi or drugs targeting caspases; scale bar represents 100 µm. (B) *In vitro* caspase 3 assay. * denotes *P* < 0.05 between *unc‐105* and wild‐type; *** denotes *P* < 0.001 between *unc‐105* and *unc‐105*; *let‐2*; one‐way ANOVA with Newman–Keuls correction. (C) Representative images of a fluorescent caspase 3 indicator substrate, Z‐DEVD‐ProRed™, from *t* = 24 h adult animals; scale bar represents 50 µm. (D) Representative images of a fluorescent caspase 3 indicator substrate, Z‐DEVD‐ProRed™, from *t* = 24 h adult animals expressing GFP localized to the mitochondria and nuclei of muscle; scale bar represents 25 µm, and the enlarged regions are an additional 2.5× magnification. Note that the GFP and red substrate combine to produce yellow/orange colour in muscle when viewed using a triple pass filter.

Having determined that degradation did not appear to be proteasome, autophagy, or calpain based, we wished to determine if degradation was due to activation of caspases. While the activation of caspases has been shown in terminally differentiated mammalian cells,^36^ this has not been an area of much study in *C*. *elegans* and caspase activation in ageing *C*. *elegans* muscle was only recently demonstrated.^37^ RNAi against either of the executioner caspases, *ced‐3* or *csp‐1*, attenuated protein degradation in *unc‐105* mutants (*Figure* 4A). Similarly, *unc‐105* mutants treated with caspase inhibitors (Quinolyl‐Valine‐Aspartic acid (QVD), Acetyl‐Aspartic acid‐Glutamic acid‐Valine‐Aspartic acid (ADEVD), and Acetyl‐Aspartic acid‐ Methionine ‐ Glutamine ‐Aspartic acid (ADMQD)) displayed reduced degradation (*Figure* 4A). Lastly, as CED‐3 exists as an inactive zymogen that is activated upon binding to CED‐4 *in vitro*,^38^ we confirmed that RNAi against *ced‐4* also attenuated degradation (*Figure* 4A). Together, these results suggest that caspases are necessary for the increased protein degradation observed in response to UNC‐105 activation. We confirmed significantly greater caspase activity in *unc‐105* mutants versus wild‐type and *unc‐105*; *let‐2* mutants (*Figure* 4B). To confirm caspase activation specifically in muscles, we fed worms a caspase substrate that exhibits red fluorescence upon cleavage. No fluorescence was observed outside of the gut in wild‐type or *unc‐105*; *let‐2* mutants, whereas red fluorescence was observed in tissue(s) outside of the gut in *unc‐105* mutants (*Figure* 4C). Next, we used worms containing Green Fluorescent Protein (GFP) expressed only in body wall muscles and found *unc‐105*, but not *unc‐105*; *let‐2* mutants display yellow/orange fluorescence, as the result of red and green fluorescence in the same tissue, in body wall muscles when fed the fluorescent caspase substrate (*Figure* 4D); note that yellow/orange fluorescence was not noted in all muscle cells at any individual time point, possibly reflective of inter‐muscle differences in caspase substrate uptake and/or degradation. Collectively, these results suggest that caspases are activated in body wall muscles in response to UNC‐105 activation and account for the increased muscle protein degradation.

### Blocking protein degradation does not substantially restore the movement defect in unc‐105 mutants

Since reducing caspase levels or activity attenuated the protein degradation observed in *unc‐105* mutants, we tested if these treatments, like *let‐2*, also attenuated the movement defect. We confirmed that *unc‐105* mutants have a significant reduction in movement in comparison with wild‐type animals at young adulthood^21^ and that this is substantially rescued in *unc‐105*; *let‐2* double mutants^15^ (*Figure* 3). However, a caspase inhibitor (ADEVD) had no effect, and *ced‐3* RNAi had only a small positive effect on movement in *unc‐105* mutants (*Figure* 3). These results combined with the observations that *crt‐1* RNAi rescues the movement defect but does not prevent protein degradation, suggest that caspase activation in *unc‐105* mutants is occurring at least somewhat independently of the movement defect.

### Activation of UNC‐105 causes fragmentation of the mitochondrial network in muscle

**Figure 5:**
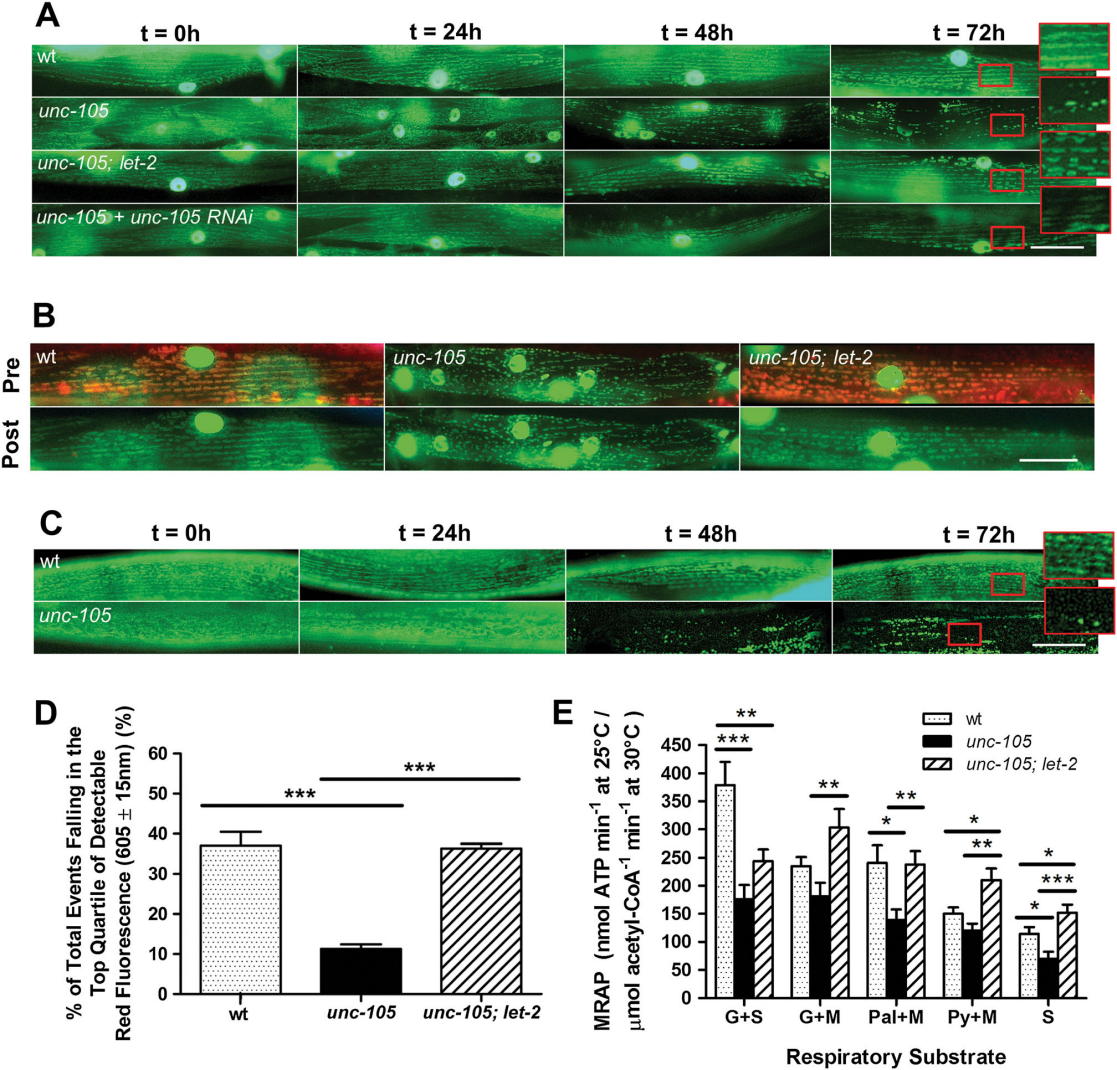
Mitochondria are dysfunctional in *unc‐105* mutants. All experiments were repeated at least three times, for (D) seven and (E) 10 independent experiments. (A–C) Synchronized young adults were used (*t* = 0 h); scale bars represent 25 µm and enlarged regions an additional 2.5× magnification. (A) Strains containing GFP localized to mitochondria and nuclei in muscle were used to assess mitochondrial architecture. (B) Strains containing GFP localized to mitochondria and nuclei in muscle were used to assess mitochondrial membrane potential specifically in muscle. Accumulation of Mitotracker® CMXRos in mitochondria in muscle as indicated both by yellow/orange mitochondria and green mitochondria in muscle post‐photobleaching. Displayed images are for *t* = 0 h young adults. (C) Worms were grown in the presence of JC‐10 to assess *in vivo* loss of mitochondrial membrane potential. (D) Loss of membrane potential was confirmed *in vitro* in *unc‐105* mutants when mitochondria were isolated from mixed populations of all three stains and were sorted using fluorescence‐activated cell sorting and JC‐1. Displayed are the percent of mitochondria showing the highest quartile of accumulation of JC‐1 as indicated by the extent of red fluorescence. (E) Measurement of maximal ATP production rates (MRAP). Displayed are data for mitochondria isolated from *n* = 250−300 mixed stage animals per sample. Substrate combinations were (G + S) glutamate and succinate; (G + M) glutamate and malate (Pal + M) palmitoyl‐l‐carnitine and malate; (Py + M) pyruvate and malate and (S) succinate. Data are expressed as a ratio to maximal citrate synthase (CS) activity; the standard marker of mitochondrial content. *P < 0.05; **P < 0.01; ***P < 0.001; one‐way ANOVA and Newman–Keuls correction.

CED‐4 binds to and activates CED‐3 *in vitro*,^38^ and inactive CED‐4 has been suggested to be localized to mitochondria.^39^ Structural damage to mitochondria occurs in HEK cells overexpressing UNC‐105^40^ and excess Na^+^ influx causes functional damage to mitochondria in mammalian muscle.^41^ Thus, because *ced‐4* RNAi blocked degradation (*Figure* 4A), we wanted to determine if mitochondria were damaged in response to UNC‐105 activation. As shown in *Figure* 5A, *unc‐105* mutants display time‐dependent fragmentation of the mitochondrial network in adult muscle. This fragmentation is prevented by RNAi against *unc‐105* and in *unc‐105*; *let‐2* double mutants (*Figure* 5A), which suggests that it is UNC‐105 hyperactivation that is causing the fragmentation.

### UNC‐105 activation impairs maintenance of the mitochondrial membrane potential

We wanted to determine if the mitochondrial disturbance was purely structural or also functional. Mitotracker® Red CMXRos is a mitochondrial dye that accumulates within mitochondria dependent upon mitochondrial membrane potential. The accumulation of Mitotracker® Red CMXRos is reduced in *unc‐105* but not in *unc‐105*; *let‐2* double mutants (*Figure* 5B), suggesting that activation of UNC‐105 results in an inability to maintain mitochondrial membrane potential. However, because Mitotracker® requires a normal plasma membrane potential in order to accumulate in mitochondria and *unc‐105* mutants have an altered membrane potential,^23^ it is possible that some or all of the reduced accumulation could be due to limited entry into muscle. Therefore, we used JC‐10, another dye that both accumulates and exits mitochondria dependent upon the mitochondrial membrane potential, thereby allowing assessment of the loss of mitochondrial membrane potential with time. When grown in the presence of JC‐10, *unc‐105* mutants display accumulation of JC‐10 in muscle mitochondria at adulthood, with loss of JC‐10 over time (*Figure* 5C). These results suggest that UNC‐105 activation causes a time‐dependent fragmentation of the mitochondrial reticulum and concomitant failure to maintain the mitochondrial membrane potential. To confirm and quantify the extent of this impairment of the mitochondrial membrane potential and determine whether it was maintained *ex vivo*, we performed fluorescence activated cell sorting of isolated mitochondria from wild‐type, *unc‐105*, and *unc‐105*; *let‐2* double mutants. In the top quartile of isolated mitochondria displaying JC‐1 accumulation in the presence of glutamate and malate, mitochondria from *unc‐105* mutants display 25% less of the potential‐dependent dye JC‐1 than wild‐type or *unc‐105*; *let‐2* animals (*Figure* 5D). These results further suggest impaired mitochondrial membrane potential in response to UNC‐105 activation.

### Activation of UNC‐105 causes a depression in the maximal mitochondrial rate of ATP production

Inability to maintain mitochondrial membrane potential will lead to declines in mitochondrial ATP production as the result of a decrease in the proton gradient that drives H^+^ through ATP synthase. Thus, we measured the maximal rate of ATP production (MRAP) in isolated mitochondria. For all substrate combinations, the mean MRAP was reduced in mitochondria isolated from *unc‐105* mutants versus wild‐type, and this reduction was significant for all substrates except glutamate + malate and pyruvate + malate (*Figure* 5E). Similarly, for all substrate combinations the reduction in mean MRAP in *unc‐105* mutants was attenuated in mitochondria isolated from *unc‐105*; *let‐2* double mutants, which was significant in all cases except glutamate + succinate (*Figure* 5E). Because MRAP is significantly depressed in *unc‐105* mutants, we determined whether glycolytic ATP production increased as a compensatory adaptation to maintain cellular total ATP production. We find no increase in maximal Phosphofructokinase (PFK) activity in *unc‐105* (*Figure* S3), which suggests metabolic compensation via non‐mitochondrial ATP production is not occurring.

### Specific mitochondrial proteins are decreased in mitochondria extracted from unc‐105 mutants

**Figure 6:**
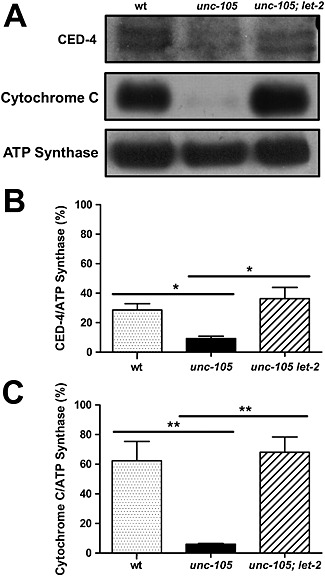
Less CED‐4 and cytochrome C are associated with mitochondria extracted from *unc‐105* mutants. All experiments were conducted at least three times. Mitochondria extracted from wild‐type, *unc‐105*, and *unc‐105*; *let‐2* double mutants were examined for mitochondrial protein content; *n* = 250−300 mixed stage animals per sample. (A) Representative western blots for CED‐4, cytochrome C, and ATP synthase. (B) Quantification of CED‐4 levels as a percentage of ATP synthase levels, *n* = 3. (C) Quantification of cytochrome C levels as a percentage of ATP synthase levels, *n* = 3. *P < 0.05; **P < 0.01, one‐way ANOVA with Newman–Keuls correction.

Having determined that mitochondria appear damaged in response to UNC‐105 activation, we tested if, as predicted by *ced‐4*‐dependent and *ced‐3*‐dependent cytosolic muscle protein degradation, less CED‐4 is associated with mitochondria following UNC‐105 activation. Mitochondria isolated from *unc‐105* mutants contain less CED‐4 and cytochrome C compared with the amount present in mitochondria isolated from wild‐type or *unc‐105*; *let‐2* double mutants (*Figure* 6). Given the reduced MRAP in *unc‐105* mutants, we also examined the amount of ATP synthase. There is no comparable decline in ATP synthase in mitochondria isolated from *unc‐105* mutants (*Figure* 6A), which confirms that reductions do not simply represent unequal loading of samples and suggests that decreased levels of CED‐4 and cytochrome C are not simply random loss of mitochondrial proteins in response to UNC‐105 activation. This observation appears to be consistent with the recent suggestion of a mitochondrial transition pore in *C*. *elegans* muscle.^42^ Additionally, our finding of less CED‐4 and cytochrome C associated with damaged mitochondria adds weight to the suggestion that CED‐4/CED‐3 dependent apoptosis, which is independent of a requirement of cytochrome C in *C*. *elegans*, reflects evolution of a lack of cytochrome C dependence in *C*. *elegans* versus evolutionary ancestors.^43^

## Discussion

### Mitochondrial dysfunction and caspase activation in UNC‐105 mutants

**Figure 7:**
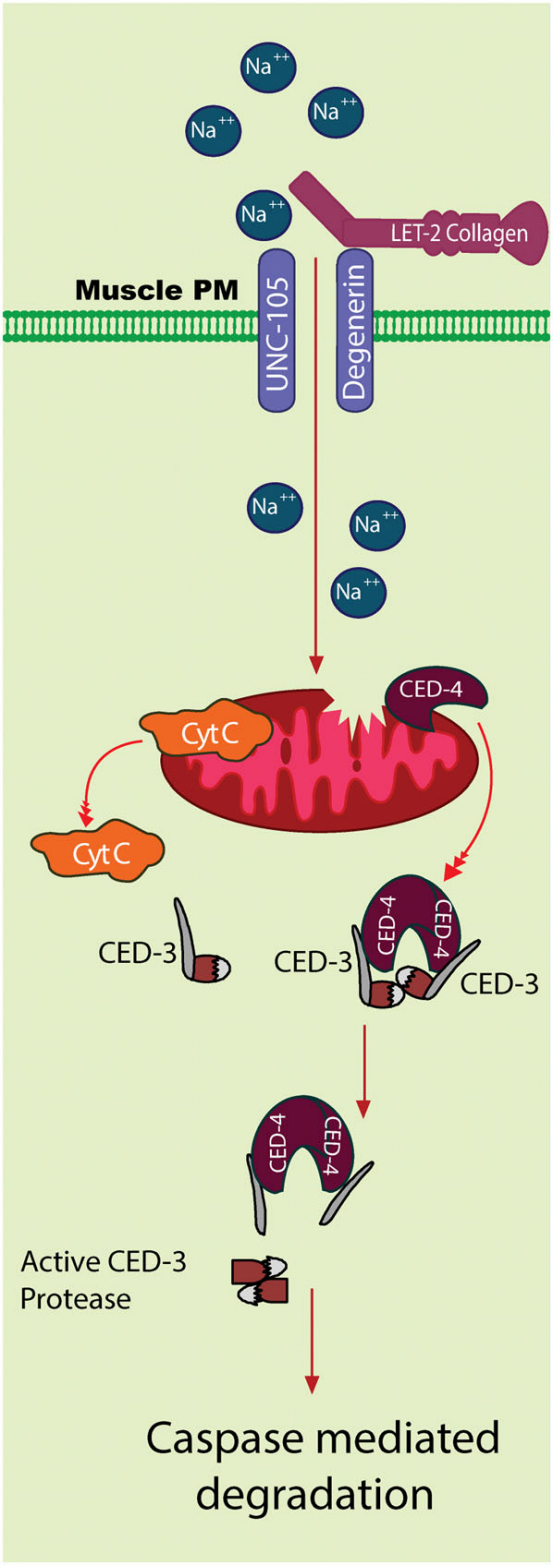
Graphical model of inferred consequences of UNC‐105 activation upon muscle mitochondria and cytosol. The UNC‐105 ion channel, when not gated by LET‐2, allows excess ion influx into muscle. In response to sustained ion influx, mitochondria are dysfunctional, and cytochrome C and CED‐4 translocate from the mitochondria. Translocated CED‐4 interacts with CED‐3 to cause CED‐3 activation. Activated CED‐3 causes cytosolic protein degradation.

We have shown that *unc‐105* but not *unc‐105*; *let‐2* double mutants display fragmentation of mitochondrial networks in muscle, decreased mitochondrial membrane potential, decreased maximal rates of mitochondrial ATP production, and reduced levels of cytochrome C and CED‐4 in extracted mitochondria. Because the second mutation in *let‐2* relieves cationic influx in *unc‐105* mutants,^23^ these results suggest that activation of UNC‐105 and consequent cationic influx into *C*. *elegans* muscle results in damage to mitochondrial structure and function *in vivo*. Additionally, because *unc‐105* but not *unc‐105*; *let‐2* double mutants display both caspase activation and pathological degradation of cytosolic muscle protein that is mediated by caspases, it appears that that activation of UNC‐105 and consequent cationic influx into *C*. *elegans* muscle results in activation of caspases in muscle cytosol *in vivo*. As CED‐4 is sufficient to activate the CED‐3 caspase *in vitro*,^38^ the findings of less CED‐4 in mitochondria extracted from *unc‐105* but not *unc‐105*; *let‐2* double mutants and RNAi against *ced‐4* attenuating degradation in *unc‐105* mutants, suggest that the pathological degradation in the muscle cytosol and the mitochondrial damage in *unc‐105* mutants are causally linked by translocation of CED‐4 away from damaged mitochondria to cause activated CED‐3 to be present in the cytosol. Combined, our data suggest a model (*Figure* 7) whereby cationic influx through activated UNC‐105 results in mitochondrial damage, CED‐4 translocation away from damaged mitochondria, CED‐4 activation of CED‐3, and subsequent pathological degradation of proteins in the muscle cytosol.

### Whole genome sequencing combined with subtractive analysis enables prospective studies of evolution in *C*. *elegans*

We used whole genome sequencing combined with subtractive analysis to identify two spontaneous mutants. We have confirmed the identity of the suppressing mutations both by known phenotypes of past suppressing mutations and by use of RNAi against each putative suppressor to phenocopy suppression of *unc‐105* phenotypes. Given the ability to perform RNAi against every gene in the genome of *C. elegans*, whole genome sequencing combined with subtractive analysis and RNAi against a single or handful of genes identified provides a rapid way of identifying and confirming suppressing mutations of physiologic interest.

### *C*. *elegans* as a model for studying genetic regulation of muscle protein degradation

Past studies have established *C*. *elegans* as a model in which genetics and genomics can be used to uncover the regulation of muscle protein degradation and revealed regulatory signals governing proteasomes, autophagy, and calpains.^3^ The present study now completes our preliminary picture of signals that regulate each of the four major proteolytic systems in *C*. *elegans* muscle. Combined, these studies enable future exploration of the relevance of these regulatory and proteolytic systems to different physiologic, pathophysiologic, or disease states.

### Relevance of mitochondrial dysfunction and muscle protein degradation to sarcopenia

Mitochondrial dysfunction has been suggested to be a hallmark of ageing. The observations that *unc‐105* mutants display disrupted sarcomeres, a movement defect, premature muscle protein degradation, and mitochondrial fragmentation and decline collectively suggest that *unc‐105* mutants may be a model of accelerated ageing (progeria). This has previously been suggested for another mutant that displays mitochondrial dysfunction.^44^ Cellular degeneration of muscle appears to be similar to that of nerves in that both appear to be a disease of age, and there is fragmentation of mitochondrial networks in response to neuronal degeneration^45^, ^46^ and in ageing muscle.^13^, ^46^ These observations coupled with the fact that *C*. *elegans* is an accepted model for studying ageing^47^ and sarcopenia^48^ suggest that future study of the role of muscle mitochondrial dysfunction and muscle protein degradation in the onset and/or progression of sarcopenia can now be achieved using *C*. *elegans*. Accordingly, it is intriguing to note that caspase activation and consequent myosin degradation in ageing *C*. *elegans* muscle have recently been reported.^37^

Sodium channel, non‐voltage gated 1 alpha subunit (SCNN1A) is a human orthologue of UNC‐105 and is expressed in human skeletal muscle (http://www.proteinatlas.org/). Given that APAF1, caspases, and mitochondria are all also expressed in human skeletal muscle, there is significant scope for our finding of excessive sodium influx into *C*. *elegans* muscle leading to mitochondrial dysfunction and caspase activation (*Figure* 7) to be relevant to human muscle function and/or pathology. If SCNN1A is mechanosensitive in human skeletal muscle, then like mechanosensitive calcium channels, it may contribute to ionic imbalance in response to stretch and in individuals with muscular dystrophy.^49^ Given that cytoskeletal proteins are common targets of caspases, there is a reason to suspect that any ionic imbalance resulting from SCNN1A hyperactivation would lead to cytoskeletal remodelling and/or dystrophy; therefore, the role of SCNN1A in human skeletal muscle would seem to warrant further investigation. Similarly, if a past report of decreased chloride transport and mitochondrial function with age in human muscle is correct,^50^ then altered ionic balance may lead to both altered mitochondrial function and cytoskeletal alterations via loss of mitochondrial membrane potential and caspase activation with age, another area that would seem to warrant further investigation. As previously noted,^50^ altered mitochondrial function with age in human muscle, as opposed to *C*. *elegans* muscle, might be an adaptation rather than a simple path to eventual cellular demise.

## Acknowledgements

This work was supported by a grant from the US National Institutes of Health National Institute for Arthritis and Musculoskeletal and Skin Diseases (AR‐054342) to N. J. S., by a grant from the Canadian Institutes of Health Research to A. R., and by a grant from Natural Sciences and Engineering Research Council of Canada to D. L. B. C. G. was funded by a Doctoral Training Studentship provided by the University of Nottingham, and all metabolic measurement consumables were funded by the University of Nottingham. The *unc‐105*(*n490*) mutant allele utilized in this work was obtained from the Caenorhabditis Genetics Center, which is funded by the US NIH National Center for Research Resources. Bioinformatic work was conducted utilizing WormBase, which is funded by the US NIH National Human Genome Research Institute and the British Medical Research Council. The funders had no role in study design, data collection and analysis, decision to publish, or preparation of the manuscript. The authors of this manuscript certify that they comply with the ethical guidelines for authorship and publishing in the Journal of Cachexia, Sarcopenia, and Muscle 2010;1:7–8 (von Haehling S, Morley JE, Coats AJ and Anker SD).

## Ethical Standards

The manuscript does not contain clinical studies or patient data. The use of invertebrate models of human disease is fully compliant with the replacement, reduction, and refinement of animal models and is therefore ethically preferred.

## Conflict of interest

None declared.

## Supporting information



Supporting info item

Supporting info item

Supporting info item

Supporting info item

Supporting info item
