# Supplementary material for: Degenerin channel activation causes caspase‐mediated protein degradation and mitochondrial dysfunction in adult C. elegans muscle
Source: J Cachexia Sarcopenia Muscle. 2015 Jun 4;7(2):181–92. doi: 10.1002/jcsm.12040 (PMC4864282; doi:10.1002/jcsm.12040)
Supplement: Supplementary file 1 — Supporting info item [file JCSM-7-181-s001.docx]

Figure S1: **Timeline of isolation and sequencing of spontaneous mutants.** Diagram showing when spontaneous mutations *xg1* and *xg2* arose in relation to the parental strain (indicated by circles) and when DNA was taken for sequencing (indicated by arrows). Points at which the parental *unc-105* strain was frozen or thawed are indicated with diamonds. Time between events is indicated in months along the appropriate line and indicates time between events on the lines. Each time line corresponds to the strain indicated at the end of the line.

Figure S2: **Schematic flowchart of analysis pipeline.** Diagram indicating how whole genome sequencing data was processed (see Materials and methods). Briefly, following sequencing reads were aligned to the *C. elegans* reference genome, SNVs were identified and categorized.

Figure S3: **Impaired Maximal ATP production rates are not compensated for by other metabolic pathways.** Displayed are data for n = 250-300 mixed stage animals per sample, each assay was conducted on 10 independent populations except Lactate with 6 independent populations. (A) Maximal citrate synthase activity, a marker of mitochondrial biogenesis. (B) Maximal PFK activity, the rate limiting enzyme in glycolysis. (C) Maximal β-HAD activity, the rate limiting enzyme of β-oxidation. (D) Total lactate, a marker of lack of usage of pyruvate by the mitochondria. * P <0.05; ** P <0.01. Data were analysed using one-way ANOVA with a Newman-Keuls correction.

**Supplemental methods (Figure S3)**

**Measurement of Metabolic Enzymes**

**Preparation of enzyme extracts and protein assays.** Animals were washed as previously described for MRAP [[14](#_ENREF_14)]. Animals underwent one freeze-thaw cycle before homogenisation. The animal pellets were resuspended in 200 µl homogenisation buffer, homogenised, and centrifuged at 24,000 g before removing the supernatant for storage at -80°C. Enzymatic assays were corrected for total protein content of the homogenisation extract, measured using the Bradford method.

**Phosphofructokinase (PFK) activity.** The analytical blank was determined by measuring 250 µl reaction buffer (0.05 M glycylglycine buffer, pH 8.2; 1 mM ATP; 1 mM fructose-6-phosphate; 0.01% human serum albumin; 14 mM cysteine and 650 μM NADH) and 20 µl homogenisation extract spectrophotometrically for 2 min at 340nm. Having measured the blank, 2 µl enzyme mix (0.4 U aldolase in glycylglycine buffer, 0.08 U triosephosphate isomerase, and 0.08 U of glycerophosphate dehydrogenase) was added and NADH was measured for 3 min at 340nm. Optimisation was completed using a positive control of 20 µl fructose-6-phosphate kinase type VII (Cat no. F0137, Sigma-Aldrich) (61 U/ml) substituted for the homogenisation extract.

**β-hydroxyacyl-CoA dehydrogenase (HAD) activity.** The analytical blank was recorded with 250 µl reaction buffer (5 ml HAD Buffer + 300 µl NADH (3 mM)) in duplicate with 15 µl homogenisation extract at 340nm for 2 min. Having read the blank, 20 µl acetoacetyl CoA was added to each well and the absorbance was read at 340 nm for a further 3 min. Samples were measured individually.
