# Supplementary figures and images for: Degenerin channel activation causes caspase‐mediated protein degradation and mitochondrial dysfunction in adult C. elegans muscle
Source: J Cachexia Sarcopenia Muscle. 2015 Jun 4;7(2):181–92. doi: 10.1002/jcsm.12040 (PMC4864282; doi:10.1002/jcsm.12040)

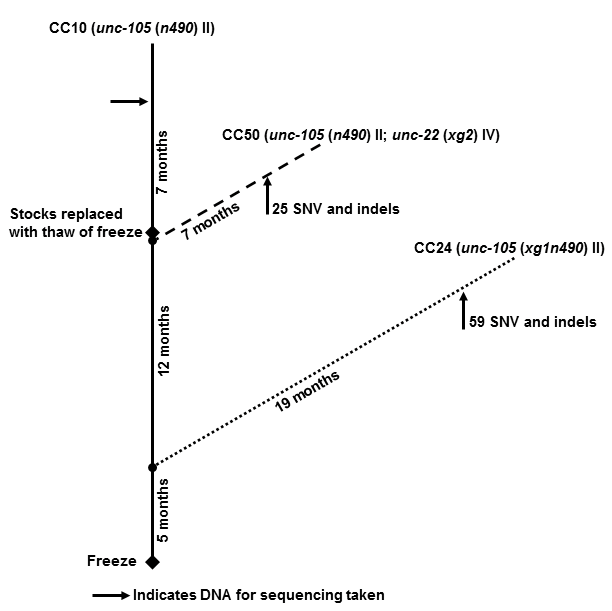

Supplement: Supplementary file 2 — Supporting info item [file JCSM-7-181-s002.tif]

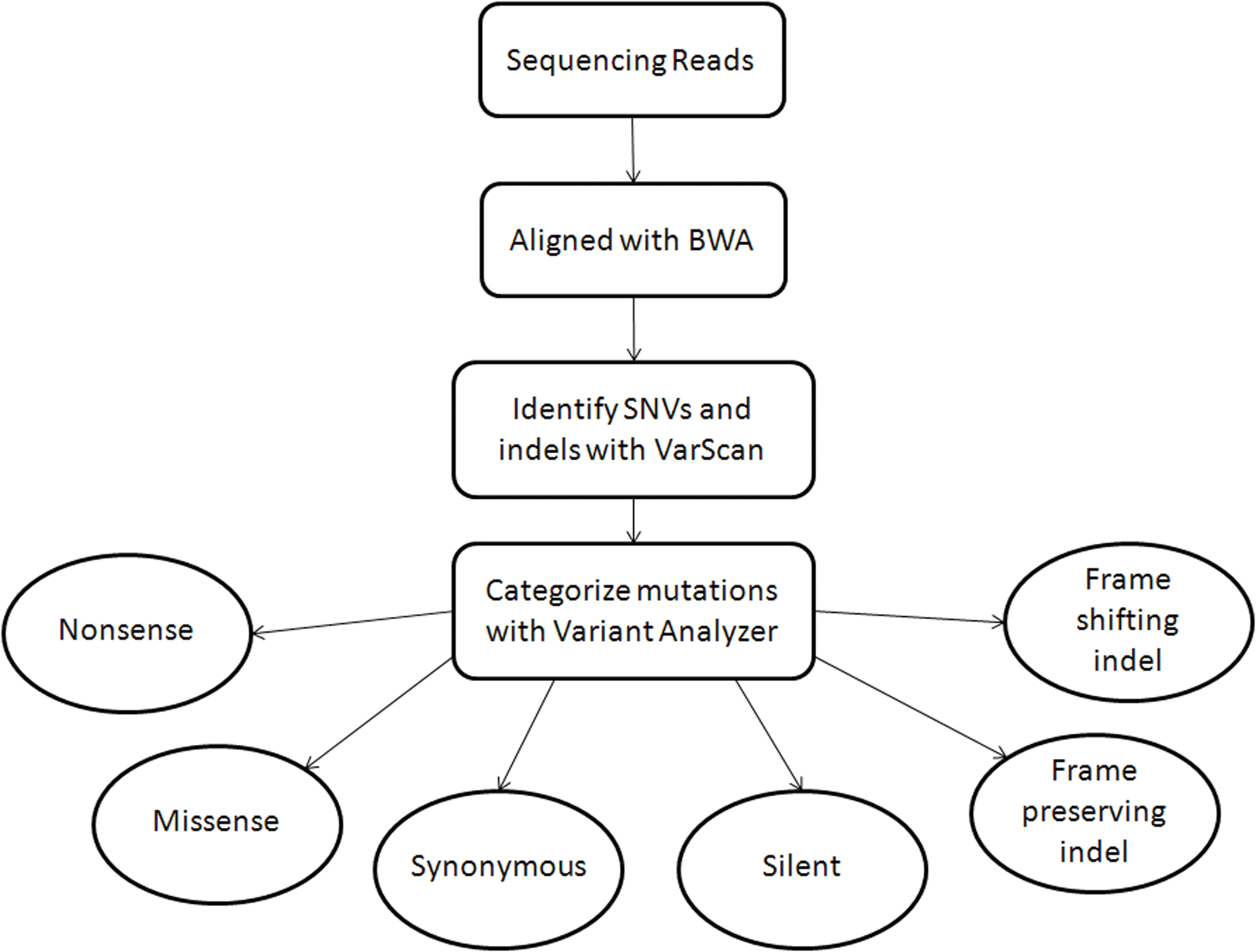

Supplement: Supplementary file 3 — Supporting info item [file JCSM-7-181-s003.tif]

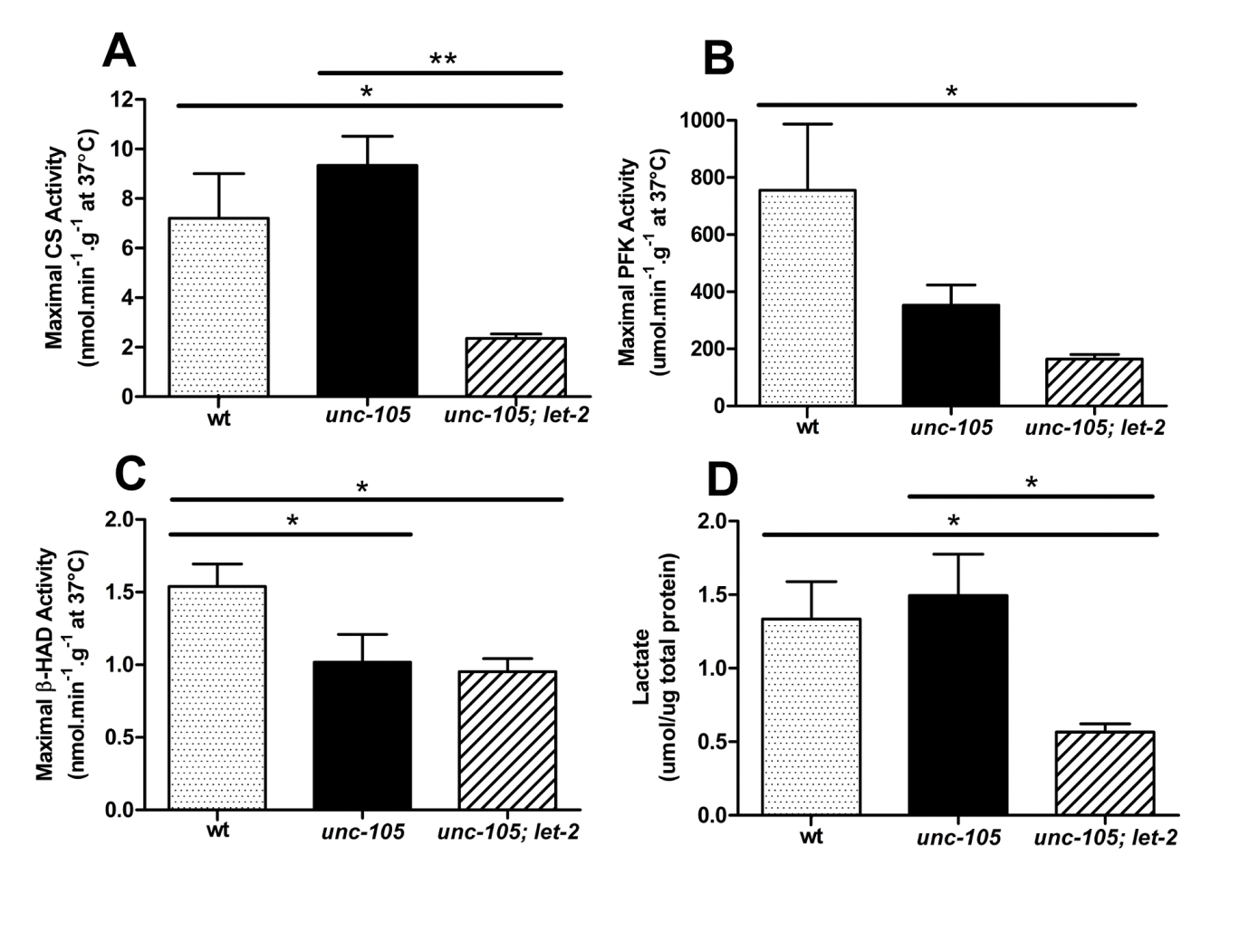

Supplement: Supplementary file 4 — Supporting info item [file JCSM-7-181-s004.tif]
